# Role of CD5/CD5L interactions in the homeostasis of regulatory lymphocyte subpopulations and the control of autoimmune disorders

**DOI:** 10.1186/1479-5876-9-S2-O6

**Published:** 2011-11-23

**Authors:** Rafael Fenutría, Vanesa G Martinez, Victor Gil, Jordi Sintes, Ines Simôes, Jesús Merino, Ramón Merino, Manel Ramos-Casals, Chander Raman, Pablo Engel, Francisco Lozano

**Affiliations:** 1 Institut d'Investigacions Biomédiques August Pi i Sunyer, Barcelona, Spain; 2 Servei de Enfermedades Autoinmunes Sistémicas, Hospital Clínic i Provincial de Barcelona, Spain; 3 Depto. de Biología Celular, Inmunología y Neurociencias, Universidad de Barcelona, Spain; 4 Depto. de Biología Molecular, Universidad de Cantabria, Santander, Spain; 5 Dept. of Medicine, University of Alabama at Birmingham, USA; 6 Servei d'Immunología, Hospital Clínic i Provincial de Barcelona, Spain

## 

CD5 is a lymphoid-specific membrane glycoprotein constitutively expressed in all T cells and a small subset of B cells, reaching its highest expression levels in T and B cells with regulatory or anergic function. CD5 is physically associated to the antigen-specific receptor in both T and B cells, negatively regulating its signalling. A soluble form of CD5 also exists which is released via proteolytic cleavage during lymphocyte activation, and whose functional relevance is unknown, in part due to the elusive nature of the CD5 ligand/s. Interestingly, circulating soluble CD5 levels appear to be increased in several autoimmune disorders, including rheumatoid arthritis and systemic lupus erithematosus. Here, we describe the generation and characterization of a transgenic mouse expressing high circulating levels of recombinant soluble human CD5 (rshCD5), aimed at studying the relevance of conserved ligand-receptor interactions mediated by CD5. Interestingly, analysis of the transgenic (rshCD5tg) mice showed a significantly reduced proportion of lymphocyte subsets with well-known regulatory properties, namely spleen Treg cells (CD4^+^CD25^+^FoxP3^+^), and peritoneal IL-10-producing CD5^+^ B cells (B10). On the other hand, NKT cells were increased in the spleen and peritoneum of transgenic mice. All of these effects were readily reproduced in wild-type C57Bl/6 mice after infusion of exogenous rshCD5 (1.2 mg/kg) for two weeks at alternate days. In agreement with these phenotypical findings, rshCD5tg mice displayed more severe forms of two different experimentally induced autoimmune diseases, such as experimental allergic encephalomyelitis (EAE) and collagen-induced arthritis (CIA) are. These data suggest that CD5 is an important immunomodulatory molecule, relevant to the homeostasis of lymphocyte regulatory subpopulations.

